# The Excessive Regulation of Early Abortion Medication in the UK: The Case for Reform

**DOI:** 10.1093/medlaw/fwab042

**Published:** 2021-12-15

**Authors:** Elizabeth Chloe Romanis, Alexandra Mullock, Jordan A Parsons

**Affiliations:** 1 Gender and Law at Durham, Durham Law School, Durham University, Durham, UK; 2 Centre for Social Ethics & Policy, Department of Law, University of Manchester, Manchester, UK; 3 Centre for Ethics in Medicine, Bristol Medical School, University of Bristol, Bristol, UK

**Keywords:** Abortion, Abortion law, Abortion regulation, Early medical abortion, Precautionary regulation, Telemedical abortion

## Abstract

Early medical abortion (EMA) involves the administration of two medications—mifepristone and misoprostol—24–48 hours apart. These routinely used medications are recognised as safe and effective by the World Health Organization which recommends this combination of medications as a safe form of abortion until nine weeks’ gestation. Despite the safety and effectiveness of this drug regimen, there exists excessive regulation around EMA. This is despite new regulations introduced in Northern Ireland in 2020 and (temporary) changes made in 2020 to allow at-home administration of mifepristone in Great Britain (following earlier changes to permit home use of misoprostol). We argue that the excessive regulation of EMA is inappropriate because it fails to recognise that abortion is essential healthcare. Further, the regulation constitutes disproportionate interference with clinical discretion and service organisation because it is medically unnecessary and prevents abortion providers in the UK from adapting their service provision in line with emerging evidence of best practice.

## I. INTRODUCTION

Early medical abortion (EMA) involves the administration of two medications—mifepristone and misoprostol—taken 24–48 hours apart, during the first 10 weeks of pregnancy.^1^ EMA is the most common abortion method in Great Britain, accounting for 73% of terminations in England and Wales,^2^ and 88% in Scotland^3^ in 2019. These medications are recognised as safe and effective by the World Health Organization (WHO), which strongly recommends this combination of medications until 9 weeks’ gestation.^4^ This highly effective drug regimen has a strong track record for safety^5^—even amongst those who self-administer without medical support.^6^ There exists a strong body of evidence that suggests EMA is ‘a safer drug regimen than an injection of penicillin’.^7^ Despite the safety and effectiveness of this drug regimen, however, there exists excessive regulation around EMA, which, we argue, is not the result of necessity to manage innate risks of these medications, but about supervising abortion decisions. In this article, we demonstrate that EMA is over-regulated in the UK. This is because of stringent requirements imposed in Great Britain by the Abortion Act 1967 (AA 1967) that require EMA to be prescribed under a strict set of conditions, specifically that it must be prescribed by doctors and requirements about *where* and under what conditions both drugs can be prescribed. The regulations in Northern Ireland,^8^ while in some ways an improvement on the AA 1967, also over-regulate EMA with requirements about who can prescribe and where the medications can be administered.

We argue that the over-regulation of EMA is inappropriate because it fails to reflect the fact that abortion is essential healthcare.^9^ Over-regulation constitutes disproportionate interference with the provision of healthcare because it is medically unnecessary and prevents abortion providers in the UK from adapting their service provision in line with emerging evidence regarding best practice.^10^ When the AA 1967 was first enacted in Great Britain, abortion was a far riskier surgical procedure.^11^ Today, however, it involves the routine administration of safe medications. The regulation is thus out-of-date. New regulations in Northern Ireland, while somewhat an ‘evolution’ of the AA 1967,^12^ continue to embody an approach that enables non-medically indicated interference. Excessive regulation across the UK perpetuates harmful narratives of people experiencing pregnancy (usually women)^13^ as being in need of supervision when it comes to reproductive decision-making and of abortion as a ‘problem’ in need of management.

This article contributes to the body of literature providing a feminist critique of the AA 1967 and calling for the decriminalisation of abortion.^14^ There is limited commentary on the regulation of abortion medications themselves (particularly mifepristone) in the UK context.^15^ This is in contrast to the USA, which has seen much discussion of the over-regulation of mifepristone by the Food and Drug Administration.^16^ Many of the concerns raised about US regulation, however, are also apparent in UK regulation. In this article, we consider the excessive regulation of EMA (in particular, mifepristone) and the extent to which over-regulation has impacted on how abortion care is delivered. While other aspects of abortion regulation are cause for concern, the over-regulation of EMA directly impacts on service-user’s experiences of care.^17^

## II. ABORTION IS HEALTHCARE

Failure to ensure access to legal abortion does not reduce the incidence of abortion.^18^ The recognition of legal abortion as a matter of public health is long-standing;^19^ the evidence ‘is clear and incontrovertible: access to safe, legal abortion on request improves health’.^20^ Indeed, the motivation behind the 1967 Act enabling legal abortion in Great Britain was to curb the high mortality and morbidity associated with clandestine abortion.^21^ The AA 1967 is often praised as a success story for having almost completely eliminated deaths resulting from ‘back-street’ terminations.^22^

The impact that access to abortion has on individual—as well as public—health is important to acknowledge. The United Nations recognises the importance of reproductive health, which it defines as ‘a state of complete physical, mental and social well-being and not merely the absence of disease or infirmity, in all matters relating to the reproductive system and to its functions and processes’.^23^ Similar recognition comes from the WHO, which acknowledges the ‘health evidence, technologies and human rights rationale for providing safe, comprehensive abortion care’.^24^ Laws that limit abortion have serious implications for female reproductive, physical, and mental health.^25^ Individuals encountering unwanted pregnancy in ‘a wide variety of medical and social circumstances experience pregnancy termination as an urgently felt need’ and as *essential* for their health and well-being.^26^ Abortion preserves health, and it can be safely provided within a health system. The Royal College of Obstetricians and Gynaecologists notes that ‘[s]afe abortions are an essential part of sexual and reproductive health; they should be an integrated component of sexual and reproductive healthcare and be available as part of routine health services.’^27^ The current legal framework, however, classifies EMA as distinct from routine health services. This disadvantages people, and their health outcomes, in various ways.

## III. EARLY MEDICAL ABORTION

EMA entails the use of mifepristone and misoprostol. Mifepristone is an antiprogestogen, meaning it inhibits the hormone progesterone—causing the breakdown of the lining of the uterus and preventing the continuation of the pregnancy. Misoprostol^28^—is a prostaglandin analogue, which causes the expulsion of the products of conception by triggering uterine contractions (not dissimilar to what occurs during spontaneous miscarriage in early pregnancy). It is possible to procure a medical abortion using only misoprostol and this is safe and effective,^29^ though it is more common to use the combination treatment because it is quicker and has fewer side effects.

The procedure is straightforward and routinely used worldwide. Mifepristone is ordinarily taken orally, but misoprostol can be administered orally, vaginally, buccally, or sublingually. While providers have preferences regarding routes of administration—often based on the latest available evidence of safety and effectiveness^30^—it is possible to offer a choice. Ideally, the two drugs are taken between 24 and 48 hours apart. They can safely be taken in quick succession,^31^ but evidence suggests that doing so increases the likelihood of side effects.^32^

EMA is widely accepted as safe, effective, and acceptable, and the WHO strongly recommends its use up to 9 weeks’ gestation.^33^ Indeed, the WHO has placed misoprostol in its Model List of Essential Medicines.^34^ Several systematic reviews clearly establish the credentials of EMA. Raymond and colleagues found that hospitalisation occurred in just 0.3% of cases, with rates of ongoing pregnancy ranging from 0% to just 3.7% (depending on dose and route of administration).^35^ More recent evidence has corroborated these findings,^36^ demonstrating that concerns about the safety and effectiveness of EMA are unfounded. While safe and effective, it must be acknowledged that EMA does come with a range of potential side effects. Some are drug related (e.g., diarrhoea, rash, and fever), and others result from the abortion process itself (e.g., lower abdominal pain). As with most drugs, side effects vary between patients. Chen and Creinin’s systematic review (which looked specifically at regimens including buccal administration of misoprostol), for example, found that between 1.9% and 61.2% of patients experiences diarrhoea across studies^37^—a significant difference. Importantly, these side effects are easily self-managed at home, with the use of over-the-counter painkillers in some circumstances. Further, in the context of patient-centred care and appropriate informed consent (which we discuss later) even a high prevalence of side effects is not cause for concern—provided patients are made aware of the risks, they should be free to accept them where the treatment aligns with their values and preferences.

High satisfaction levels in patients undergoing EMA have consistently been reported. One systematic review found satisfaction to range from 72% to 99%—incidentally, satisfaction was higher among those who were able to undergo the treatment at home rather than in a clinic.^38^ Acceptability has also been demonstrated across qualitative studies. One participant in a study on the Thailand-Burma border (a misoprostol-only regimen) remarked that it was ‘just like having a period with back pain’.^39^ Patients often comment on privacy and convenience as positive aspects of the overall treatment process including that the ability to complete the procedure at home was ‘relieving’ because it is an environment in which they feel ‘safe and comfortable’.^40^

As EMA is a demonstrably safe and effective drug treatment with easily managed side effects, it is clearly clinically unnecessary and inappropriate to treat these drugs differently from other prescription medicines. Excessive interference instead embodies *political* concerns about abortion itself as opposed to the safety of the procedure.

## IV. THE LEGAL FRAMEWORK

Regulation of EMA is at odds with government and healthcare regulators’ policy within the context of pharmaceutical regulation. There is excessive intervention by the law into clinical discretion in prescribing and advising on the use of mifepristone and misoprostol compared to other medications. This makes abortion harder to access than other forms of routine healthcare, which constitutes an unjustified interference with people’s health.

The Medicines Act 1968 requires that ‘prescription-only medicines’,^41^ encompassing mifepristone and misoprostol,^42^ must only be supplied by the prescription of an appropriate practitioner and administered as per the practitioner’s directions. The requirement that some drugs are available only by prescription exists to ensure that appropriate expertise is deployed where without it there is the potential for harm. Doctors ‘are empowered by governments to limit patients’ access to medicines out of a concern that patients with unrestricted access would otherwise make inadvisable treatment decisions, or abuse potentially dangerous medicines’.^43^ A patient has no right to a prescription. The patient can request particular medications, but if the doctor (or other qualified health care professional) believes that the medications are not clinically indicated, or may be misused, the patient will likely leave the consultation ‘empty-handed’ as ultimately ‘patients do not decide which drugs they are prescribed’.^44^ By virtue of regulation 214 of the Human Medicines Regulations 2012, professionals deemed qualified to prescribe a prescription-only medicine include (among others) doctors, nurse independent prescribers, and pharmacist independent prescribers. These professionals act as ‘gate-keepers’ to medicines.^45^ The general conditions placed on prescription in the regulations are that:

- it must be signed in ink by the prescribing practitioner;^46^
- the contents of the prescription must be indelible;
- the prescription must contain the date on which the prescription is signed or the date on which it can be dispensed to the patient, identifying information about the practitioner including their working address, the name and address of the patient, and the patient’s age if they are under 12.^47^

Where the prescription is provided electronically, conditions (a) and (b) need not be met, but the prescription must be signed with an ‘advanced electronic signature’^48^ and sent directly to the professional who will dispense it.^49^ There is, in these regulations, no requirement that this practitioner has examined the patient to make a prescription, nor are there any other specific requirements in the law relating to the *circumstances* of prescription.^50^ In contrast, however, there are numerous legal rules surrounding the circumstances of abortion medications. In this section, we outline the legal framework surrounding abortion (and consequently EMA) in the UK. There are some differences in regulation across the UK, as the legality of abortion differs between jurisdictions (England and Wales, Scotland, and Northern Ireland).

The administration of abortion medications in England and Wales remains a criminal offence by virtue of the Offences Against the Person Act 1861 (OAPA 1861), which renders it an offence for a person to ‘unlawfully supply or procure any poison or any other noxious thing … knowing that the same is intended to be unlawfully used or employed with intent to procure the miscarriage of any woman’.^51^ The OAPA 1861 does not apply in Scotland, but the administration of abortion medications is criminal by virtue of the common law.^52^ Abortion medications can only be lawfully administered in compliance with the provisions of the AA 1967. This imposes a strict set of requirements on the administration of abortion medications in Great Britain, including specifying the circumstances in which prescription is appropriate,^53^ specifying who can prescribe the medications,^54^ and specifying *where* the medications can be prescribed and administered.^55^

While the provisions of the OAPA 1861 that criminalised abortion applied in Northern Ireland (until recently), the AA 1967 did not.^56^ Until 2019, abortion—at any point in pregnancy—could only be performed in Northern Ireland (on the grounds that it would not constitute unlawful miscarriage) where necessary to save the pregnant person’s life.^57^ In 2019, abortion was *partially* decriminalised as the relevant provisions of the OAPA 1861 were repealed by the Northern Ireland (Executive Formation etc) Act 2019.^58^ The AA 1967 never applied in Northern Ireland, which meant that there was no law concerning abortion before viability in Northern Ireland until new regulations were enacted in 2020. The Abortion (Northern Ireland) (No. 2) Regulations 2020 were introduced to regulate abortion on similar grounds to the AA 1967, though with some significant differences. The most notable is that the regulations create a new criminal offence—the provision of abortion outside the terms of the regulations.^59^ This is an offence that is only committed by persons other than the pregnant person themselves,^60^ who are subject to no criminal sanction when they abort in circumstances beyond those determined permissible.

The AA 1967 is woefully out-of-step with modern day abortion provision—specifically the realities of EMA prescription and administration. Although in some ways the Northern Ireland regulations are, on paper, more permissive and reflective of the realities of modern abortion care, there is still much to be desired in their regulation of where abortions can take place, and their implementation in practice.

### A. Specifying the Circumstances for Appropriate Prescription

For abortion to be lawful, it must be provided on one of the *clinical* grounds provided in section one of the AA 1967.^61^ Pregnancy may be terminated where two medical practitioners form the opinion in good faith that:

- the pregnancy has not exceeded 24 weeks and continuance of the pregnancy involves greater risk than if the pregnancy were terminated of injury to the physical or mental health of the pregnant person or any existing children of their family;
- termination is necessary to prevent ‘grave permanent injury to the physical or mental health’ of the pregnant person;
- continuance of the pregnancy involves a greater risk to the pregnant person’s life than termination of pregnancy; and/or
- there is a substantial risk that ‘if the child were born it would suffer from such physical or mental abnormalities as to be seriously handicapped’.^62^

The Act instructs doctors to take a person’s ‘actual and reasonably foreseeable environment’ into account when making decisions about risk of injury to health.^63^ The Act does afford significant discretion to doctors, such that it is often described as making all early pregnancies ‘legally terminable’,^64^ because it is interpreted broadly.^65^ Early in pregnancy, abortion will always be *statistically* safer than carrying pregnancy to term and birthing.^66^ It is easy to justify prescription of EMA under the AA 1967 because continuing the pregnancy is more detrimental to the patient than terminating it with medication.^67^ Risk-benefit analysis supports the position that EMA is safe. We have outlined the mifepristone-misoprostol regimen’s safety credentials. Weighing EMA against not accessing abortion (and therefore being forced to birth) supports the comparative safety of the treatment.^68^ Despite the law rendering EMA easily *permissible*, it is nevertheless incredibly prescriptive about when abortion can be provided (and thus when abortion medications can be prescribed). There is no other law that, in this way, significantly interferes with clinical discretion by specifying *the circumstances in which* prescribing a particular treatment is appropriate.

The AA 1967 can be contrasted with the 2020 regulations in Northern Ireland, which has provision for abortion on similar grounds. Regulation 4 specifies that where the pregnancy has not exceeded 24 weeks, abortion can be lawfully provided where two medical practitioners form the opinion in good faith that ‘continuance of the pregnancy would involve risk of injury to the physical or mental health of the pregnant woman which is greater than if the pregnancy were terminated’.^69^ However, under regulation 3, abortion can also be provided where a medical practitioner forms the opinion in good faith that the pregnancy has not exceeded 12 weeks with no further caveats. Before 12 weeks, there is no interference with the clinical discretion of the medical practitioner in making a prescription. On this count, the abortion regulations in Northern Ireland are—in no small way—an evolution of the AA 1967. Though the same criticism regarding the interference with clinical discretion still applies to the legal status of abortion performed after 12 weeks’ gestation.^70^ The law in Great Britain (and Northern Ireland to a lesser extent) interferes with clinical discretion in prescribing mifepristone and misoprostol by specifying when prescription is appropriate.

### B. Specifying Who Can Prescribe These Medications

Prescription-only medicines can be provided by a variety of healthcare practitioners qualified to handle medications. However, additional requirements are placed on abortifacients when used for abortion. The AA 1967 specifies that only *medical practitioners* can perform abortions. This does not mean that they necessarily must perform/supervise every aspect of treatment. The House of Lords stipulated that what the Act requires is that ‘a registered medical practitioner, whom I will refer to as a doctor, should accept responsibility for all stages of the treatment for the termination of the pregnancy. The particular method to be used should be decided by the doctor *in charge of* the treatment for termination of the pregnancy’.^71^ While National Institute of Health and Care Excellence (NICE) guidance recommends that providers of abortion care ‘maximise the role of nurses and midwives in providing care’^72^ it remains the case that a doctor is ‘in charge’. It must be a doctor, with the second opinion of another doctor certifying this action, who prescribes EMA—meaning quite literally providing the dispensing certificate and written instructions about how the patient is to administer the medications. There is no other medical procedure that—by virtue of legal requirements—means that two doctors must provide their signature before it can be performed.^73^ That it is a doctor who must prescribe (and specifically after *two* doctors have agreed this is the person's circumstances fit within the socio-medical ground for abortion) sets apart EMA from other prescription-only medications.

In Northern Ireland, by contrast, and with the importance of ‘task sharing’ in abortion care^74^ appropriately recognised, the 2020 regulations stipulate that it is registered medical *professionals* that can provide or supervise abortion.^75^ This term was deliberately adopted to be inclusive of registered nurses and midwives^76^ deemed qualified to prescribe any prescription-only medications, but equally to be exclusive of other groups of persons who might be deemed qualified to prescribe in other contexts. The explanatory memorandum explains that ‘registered medical professional’ was adopted because ‘other terms can include a wider group of practitioners or healthcare professionals, for example, psychologists or pharmacists’.^77^ EMA is still, though to a lesser degree than in Great Britain, distinguished from other medications.

### C. Specifying where Abortion Medications Can Be Prescribed and Administered

In addition to the AA 1967’s requirements that EMA prescriptions are provided on particular grounds and by doctors, the Act also has traditionally placed limitations on *where* EMA can be administered following prescription. These laws were (temporarily) amended in Great Britain in 2020 in response to the COVID-19 pandemic,^78^ and at the time of writing/revising we await news about whether these changes will be made permanent following public consultations undertaken in late 2020/early 2021.^79^ Changes to relax restrictions on where EMA can be administered were not introduced in Northern Ireland at this time.

The AA 1967 affords relevant government ministers in Great Britain the power to specify where EMA can be administered.^80^ Further, within the power to approve a place for the purposes of the performing of abortion (including EMA administration) ministers have the authority to specify *how* the use of medicines is carried out.^81^ Not only can relevant government ministers determine *where* EMA may be appropriately administered—they can also provide detailed conditions about *how and when* the medications would have to be administered for that place to be suitable. That the power extends to dictating the circumstances of administration has been affirmed by the Scottish Court of Session^82^ and the Court of Appeal.^83^ Where the medications are not prescribed or administered in a way compliant with the given conditions this would be a commission of a criminal offence under the OAPA 1861—on the part of both the pregnant person and the prescribing doctor.^84^ Similar provision is made in the Northern Ireland regulations, which specify that the Department of Health may approve a place for abortion including, in relation to EMA, ‘the use of such medicines as may be specified in the approval’ such that the administration of EMA in that place would be lawful only where carried out in the manner specified.^85^ A doctor who prescribed medication in a manner not compliant with the given conditions (and a non-emergency) would be guilty of a criminal offence under the regulations.^86^

These powers, in Great Britain and Northern Ireland, afford politicians considerable power to interfere with the clinical discretion of doctors because they can dictate, using approval orders, the exact circumstances of how a prescription is, for example, issued and dispensed, and the instructions for administration. That interference is enforced with potential criminal sanction.

#### 1. Pre-2020 Prohibition on Home Use of Mifepristone

In December 2018, Matt Hancock, Secretary of State for Health and Social Care, issued an approval order declaring that, ‘the home of a pregnant woman who is undergoing treatment for the purposes of termination of her pregnancy is approved as a class of place where the second stage of treatment for termination of pregnancy may be carried out where the treatment is carried out in a manner specified [in England]’.^87^ Termination was described as lawful where;

- ‘the pregnant woman has attended a clinic where she has been prescribed Mifepristone and Misoprostol to be taken for the purposes of termination of her pregnancy; and
- the pregnant woman has taken the Mifepristone at the clinic, wants to carry out the second stage of treatment at home and the gestation of the pregnancy has not exceeded nine weeks and six days at the time the Mifepristone is taken’.^88^

This approval order sets out exactly the same procedure for prescription as the approval order issued by Scottish Minister for Public Health, Aileen Campbell, a year earlier,^89^ and by Welsh Minister for Health and Social Services, Vaughan Gething, in June 2018.^90^ The effect of all these declarations was that it became lawful for a person to self-administer misoprostol at home, however it remained *unlawful* to take mifepristone at home. It also was *unlawful* for a practitioner to prescribe both abortion medications anywhere except a hospital or clinic. The order specified in explicit terms that the clinic is *where* the person is prescribed EMA (here meaning the production and signing of the written document confirming the appropriateness of the medications and dispensing instructions). In a challenge to the Scottish approval order raised by the Society for the Protection for Unborn Children,^91^ Lady Wise summarised the four conditions necessary for ‘at home’ administration of misoprostol to be lawful:

- ‘First, that she is ordinarily resident at the home in question,
- Secondly, that she wants to carry out the stage of the treatment that involves the taking of Misoprostol at that ordinary residence,
- Thirdly, that she does so having been prescribed both Mifepristone and Misoprostol at a clinic, and
- Fourthly, that she has taken the Mifepristone at that clinic’.

There was, in these approval orders, no requirement that *consultation* take place in a clinic. NICE guidelines have for some time recommend in their Abortion Care guidance that doctors ‘consider providing abortion assessments by phone or video-call for women who prefer this’.^92^ This remote consultation would be lawful only to advise a person about their options for accessing treatment or to provide a referral to an approved place to access treatment. Thus, it was lawful for doctors to make determinations about access to treatment via phone, online, or video-link, but they could not lawfully *prescribe* (meaning generate the dispensing instructions for the medications and sign it) in this interaction.

In Northern Ireland, the 2020 regulations set out lawful grounds for home use of mifepristone. A pregnant person can administer misoprostol in their home (their permanent address/where they usually reside in Northern Ireland)^93^ where they have attended a hospital or clinic ‘where she has been prescribed mifepristone and misoprostol to be taken for the purposes of terminating the pregnancy’; has taken mifepristone in that place and their pregnancy has not exceeded 10 weeks.^94^ Before abortion was partially decriminalised in Northern Ireland in 2019,^95^ law enforcement adopted a punitive approach in responding to individuals who procured abortifacients from unlawful online sources (such as Women on Web).^96^ There is now no criminal sanction in Northern Ireland for pregnant people who obtain abortifacients through informal (unlawful) channels;^97^ however, a medical practitioner who prescribes medications in a manner non-compliant with the conditions/process outlined in regulation would be guilty of a criminal offence.^98^

#### 2. Temporary Changes In 2020—Relaxation Allowing Home Use of Mifepristone

In response to the COVID-19 pandemic making access to abortion clinics more difficult,^99^ and considerable campaigning by public health and reproductive health specialists, approval orders were issued in late March 2020 across Great Britain to allow home administration of *both* abortion medications. The first order was issued on 30 March 2020 in England, which stipulated that, until such time that the Coronavirus Act 2020 ceases to apply or until 30 March 2022 (whichever is sooner), both mifepristone and misoprostol could be administered at home under the following conditions:

- The pregnant person must have had a consultation with an approved place via video-link, telephone conference, or other electronic means, or had a consultation with a registered medical practitioner by electronic means; and
- They must have been prescribed both drugs for the purposes of abortion; and
- They must not be more than nine weeks at six days pregnant at the point medications would be administered; and
- They must administer the medications in the place in England where they are ordinarily resident.^100^

The significant changes are that prescription need not take place in a particular place (so the dispensing instructions can be issued and signed without the pregnant person needing to be in the clinic at the time this is produced), and that both medications can be administered in the pregnant person’s home. An approval order that mirrors this was issued in Wales the following day (31 March 2020) with the same conditions for pregnant people administering medications at home (the place they are ordinarily resident in Wales) and with the same sunset clause (expiration with the repeal of the Coronavirus Act 2020 or 31 March 2020—whichever is sooner).^101^ Similar changes were made in Scotland on 31 March 2020^102^ with some notable differences from those issued in England and Wales. First, there is no expiration date on the approval order, although the explanatory note from the Chief Medical Officer does indicate that it will be revoked in future.^103^ Second, the approval order does not specify a gestational limit for home use of both medications, but the explanatory guidance suggests a maximum of 11 weeks and six days. Far more clinical discretion is afforded here.^104^ Finally, the Scottish order specifies that consultation must be by telephone or video-link (without the caveat of ‘or any other electronic means’ like in England and Wales).^105^ EMA prescribed in these conditions must also be administered at the address in Scotland where the person is ordinarily resident.^106^ Notably, in all three jurisdictions the home remains restrictively defined,^107^ which can result in access issues for people who do not have permanent homes, for example, people in temporary shelters or foster care.^108^

In Northern Ireland, in contrast, no change has been made to allow home use of mifepristone (despite the Northern Irish Department of Health having the power to do so).^109^ People in Northern Ireland must still attend a clinic to be prescribed EMA and be supervised self-administering mifepristone.^110^ Since there have been public consultations in Great Britain about these changes to the law, organisations providing abortion care hope that the changes might become permanent. Northern Ireland ought to follow suit, as the failure of the Northern Irish Department of Health to act has resulted in substantial inequality in access to care between Northern Ireland and the rest of the UK—it is far more difficult for people in Northern Ireland living in rural areas, who are socio-economically disadvantaged or are otherwise vulnerable, to access care compared to counterparts in Great Britain. However, with the track record of Stormont and the position of the current Health Minister, Robin Swann, this is unlikely to happen in the near future.

Parliament recognises that some medications should be prescription-only (as opposed to being ‘over-the-counter’) as a means of limiting access to those circumstances in which their use is safe and clinically advisable.^111^ They have delegated the decision-making power to doctors. They do not regulate beyond specifying that some medications must be prescribed, because it is a matter of clinical discretion when a medication is clinically indicated and safe. We have described the detailed conditions in the regulations regarding where prescriptions can be issued and the circumstances in which doctors (and nurses/midwives in Northern Ireland) can direct the administration of EMA. Such regulations are not made in respect of other prescription-only medicines that are not additionally recognised as controlled substances per the Misuse of Drugs Regulations 2011.

## V. EMA REGULATION IS CLINICALLY UNWARRANTED

As Cook and Erdman explain, ‘human rights standards define a law as arbitrary if it inflicts harm without need or reason, or if its prohibitions bear no connection to or undermine its aims, however legitimate’.^112^ In this section, we demonstrate how the excessive regulation of EMA—relative to other prescription-only medications—is arbitrary because there is no evidence to justify such requirements. We focus on two key restrictions—the pre-2020 prohibition on the home use of mifepristone in Great Britain (and continued prohibition in Northern Ireland), and the prohibition on nurse prescription in Great Britain. These are the two most significant areas in which EMA is ‘singled out’ by the regulatory regimes in Great Britain and Northern Ireland.

We start with remote prescription of abortion medications. Remote prescription is related to, but distinct from *home use* of abortion medications. Telemedical/remote provision of abortion medications exists on a spectrum of options.^113^ Home use is just one aspect of a remote care pathway—it literally entails a person taking abortion medications at home—but it is plausible that the person still attended a clinic in person for a prescription. In 2020 in Great Britain, a *fully* remote care pathway was (temporarily) enabled because doctors have been permitted to prescribe abortion medications without face-to-face consultations (remote prescription), and patients have been permitted to administer mifepristone at home (home use). Both remote prescription and home use are important to improve access to abortion, but the distinction is important to draw when examining the clinical necessity of interference into the prescription and administration of abortion medications.

Since fully remote care has been temporarily lawful (at the time of writing, for 18 months) a wealth of data now demonstrates the safety of home use of mifepristone (in addition to misoprostol). One study compiled data from the new TEMA services established by the three largest abortion providers in England.^114^ Comparing the new TEMA services with the providers’ existing in-person care pathways, this study found comparable results in terms of treatment success, serious adverse events, and incidence of ectopic pregnancy.^115^ Notably, the mean waiting time for treatment was shorter in the TEMA group by 4.2 days,^116^ which speaks to concerns that care might be accessed later in pregnancy. Another study examined the TEMA service established by NHS Lothian in Scotland, and similarly found the treatment to be safe—just 0.3% of patients were admitted to hospital with haemorrhage (neither requiring transfusion), with other unscheduled hospital visits largely prompted by pain and bleeding requiring observation only.^117^ These more recent studies add to the body of evidence establishing that remote prescription and home use of abortion medications is safe.^118^ However, the law remains set to revert to requiring face-to-face prescription and supervision of mifepristone swallowing in Great Britain,^119^ and this remains the case in Northern Ireland. Given the safety of remote prescription and home use, there is no reason to insist that the law revert to a position of people having to self-administer mifepristone in clinics rather than at home.

There may, however, be a livelier debate about the suitability of *remote prescription* of EMA in non-emergency circumstances. The argument might be made that patients should attend clinics to be prescribed EMA, and then they could self-administer at home. To establish that abortion needs to be singled out as a treatment for which remote prescription is not appropriate, it must be established that—outside of a pandemic—either there is a need for a physical examination to prescribe and supply mifepristone and misoprostol, or that there is a need for a face-to-face consultation because it is *not possible* to provide adequate information via remote consultation. In this section, we also examine the prohibition of nurse prescribing in Great Britain—which, again, we consider arbitrary regulation. To establish that nurse prescription of EMA is impermissible, it would need to be demonstrated that nurses are not qualified to make such prescriptions, that prescription by a doctor is necessary to preserve the health of those receiving EMA. If none of these claims related to remote prescription or nurse prescription can be successfully made, then the law is clearly disproportionate to its aim; providing access to safe abortion.

### A. Should There be a Requirement for Physical Examination to Prescribe Mifepristone and Misoprostol?

There is widespread acceptance that prescription can be perfectly appropriate without examination or face-to-face consultation. The General Medical Council (GMC) specifies that remote consultation and prescription can be appropriate where:

- ‘The patient’s clinical need or treatment is straightforward
- You can give patients all the information they want and need about treatment options by phone, internet or video-link
- You have a safe system in place to prescribe
- You have access to the patient’s medical records
- You don’t need to examine the patient
- The patient has capacity to decide about treatment’^120^

Since 2013 the GMC’s guidance, *Good practice in prescribing and managing medicines and devices*, has acknowledged that prescription without examination is routine. The 2013 guidance specified that doctors can prescribe by telephone, online, and by video-link where they have ‘adequate knowledge of the patient’s health, and are satisfied that the medicines serve the patient’s needs’, considering the limitations of the medium of communication, whether there is a need for physical examination, and whether there is access to the patient’s medical records.^121^ It further specifies that in order to prescribe remotely doctors must satisfy themselves that they can ‘make an adequate assessment, establish a dialogue and obtain the patient’s consent in accordance with [GMC] guidance’.^122^ Updated guidance issued in 2021^123^ is more comprehensive—likely due to the increased use of remote prescription since the pandemic. It recommends that face-to-face consultation may be more appropriate where there is uncertainty about patient capacity, where physical examination is necessary, where there is limited access to patient records (especially relevant where the medication requires additional safeguards), where there is concern that a patient either does not have a safe and confidential place for remote consultation, or there is a concern that they may not be able to make a free decision (because of abuse or coercion).^124^

The legal requirement that there is a face-to-face interaction to prescribe abortion medication does not incorporate a legal requirement to conduct a physical examination in order to prescribe, though the argument might be made that the reason face-to-face prescription is dictated is *so that* a physical examination is undertaken. The law is silent generally as to exactly where physical examination is necessary to prescribe,^125^ and dictation to this effect would be overstepping into the realm of clinical discretion. As a matter of fact then, is there evidence to suggest that a doctor must undertake a physical examination of a pregnant person in order to prescribe abortion medication? If this question is answered in the negative, it means that this aspect of the GMC guidance about when remote consultations are inappropriate cannot be relied upon to differentiate abortion medications from other prescriptions that are routinely, as a matter of good medical practice, prescribed remotely after teleconsultation. Physical examination is not necessary because there are no risks associated with either of these medications that are markedly better managed by a physical examination *before* the medications are prescribed and provided to patients except in exceptional circumstances.

It might be argued that the importance in requiring a pregnant person to attend a clinic in person is to confirm that they are pregnant before they receive treatment for abortion. Such a requirement labels pregnant people as in need of supervision to diagnose and ascertain their symptoms. While there may be many instances in which healthcare professionals may prefer to see a patient prior to a prescription, this is a matter of *clinical discretion* rather than a legal requirement. That the law is intervening to make clinic attendance compulsory is setting pregnancy apart as a condition that necessitates special oversight. It is wrong to approach healthcare from the starting point that a pregnant person does not understand their condition. This is potentially commonplace in the healthcare setting in that pregnant people (usually women) are seemingly distrusted in self-reporting. This is a particularly gendered phenomenon—for example, women frequently struggle to get their endometriosis symptoms recognised and appropriately diagnosed.^126^ People seeking access to termination of pregnancy are capable of ascertaining that they are pregnant (because of the availability of over-the-counter accurate pregnancy tests) and deciding that termination is appropriate for them. The concern more often raised that even where an individual can identify that they are pregnant, they might not be well placed to accurately date their pregnancy, and therefore a physical examination (usually an ultrasound scan) should be a mandatory part of the prescription of abortion medication. Notably, however,—even before COVID-19 resulted in sweeping changes in 2020—there were already moves away from the use of ultrasound scanning before the prescription of EMA. In the USA, the FDA removed the requirement for a physical examination before the prescription of mifepristone from their ‘Elements for Safe Use’ protocol for the drug in 2016.^127^ Legal prescription of mifepristone was never conditional on the performance of an ultrasound in Great Britain, but it was routinely provided by abortion providers. In early 2020 (before the pandemic), however, BPAS began trialling ‘no-touch termination’ in their clinics—prescription without ultrasound/any physical examination.^128^

Where there is no ultrasound or physical examination, gestational age is ordinarily ascertained based on the patient’s menstrual history. This requires pregnant people to reliably recall this information with accuracy—something which evidence suggests is appropriate. Bracken and colleagues have studied this by comparing ultrasound results with patient estimates (based on menstrual history) of their gestational age, identifying how many fall within the ‘caution zone’ (instances where the ultrasound determined the gestation to be 63 days or more but the patient estimated it to be below 63 days).^129^ In this study, just 2.4% of participants fell within the ‘caution zone’^130^—meaning they could have been offered treatment past the gestational limit (assuming, i.e., that the limit is 63 days) in the absence of ultrasound scanning. Given that the gestational limit in England and Wales is 9 weeks and 6 days, it is worth noting that only 0.8% would have been within the ‘caution zone’ if the results are adjusted to this limit.^131^ Similar results were found in a more recent systematic review,^132^ demonstrating that, for the most part, pregnant people date their pregnancies based on menstrual history within an acceptable margin of error.

The claim might also be made that some people may *deliberately mislead* a consulting healthcare professional about the duration of their pregnancy in order to obtain EMA. In one 2020 study conducted in Great Britain, only 0.04% of service users were found to be past 10 weeks’ gestation.^133^ If misreporting is occurring (this study does not indicate whether the individuals over the 10 week limit *knew* that they were), it is an extremely rare occurrence. That an individual could misreport does not speak to the overall safety of remote prescription.^134^

It has been argued that that physical examination should be undertaken to confirm pregnancy and ensure that people are not attending clinics to access treatment on behalf of others;^135^ effectively passing their prescription onto someone else. Jackson has suggested that, at least in some instances of private virtual consultations, ‘without seeing the patient, there must be doubts about whether their condition can be adequately assessed online, especially given how easy it is to disguise one’s identity on the internet’.^136^ First, we suggest that the likelihood of people obtaining medications to give them to others is very low. Furthermore, such an objection is an instance of regulation assuming that people with the physiology to become pregnant are irresponsible users of healthcare. *If* there were some instances of this occurring it would only be in the tiny minority of cases and is insufficient to deprive the majority of a service that drastically improves access. There is no reason to suppose an examination would be necessary to confirm a patient is pregnant—and it is an instance of testimonial injustice to assume that a person’s status as pregnant needs verification beyond their confirmation. The important point here is that stigma is perpetuated *in the law* where the face-to-face requirement exists, as it is not levelled against other medications.

So, at best, the argument would be that a face-to-face consultation (rather than a physical examination) would be necessary in advance of prescription. However, similar concerns apply to the prescription of any medication whether it is prescribed virtually or face-to-face. As Jackson has also noted, while the requirement for prescription means in theory that we can ensure medicines are taken only by persons for whom it is clinically indicated and although ‘labels on prescription drugs specify that the medicine should be taken only by the person for whom it was prescribed, in practice it is impossible to exercise much control over the bottle or packet of pills once the person has taken it home’.^137^ Concerns about the obtaining of medications for others are often levied against the provision of large supplies of birth control in GUM clinics despite the lack of evidence that people share the contraceptive pill, since people want to consult with medical practitioners about their options and what is best for *them*. People using remote consultation services to access abortion medications for others is very unlikely, as abortifacients are already easily accessed unlawfully online,^138^ and remote consultation will better allow people who might otherwise be afraid to attend a clinic to consult an appropriate practitioner to do so. People who might otherwise have obtained, or asked a friend to obtain, abortion medications unlawfully are more likely to want to engage with these services for themselves as their confidentiality will feel better protected in accessing formal services (not least because there would not be a risk of criminal sanction). Indeed, those accessing abortion care through BPAS can request that information about their treatment is not shared with their GP,^139^ which may put at ease those who are concerned about, for example, their family finding out about an abortion.

The fact that *some* people could or might abuse services inappropriately should not be used as an excuse to prevent others from accessing their healthcare in a way that is beneficial for them.^140^ This same point might be made about the prescription of antibiotics, antidepressants, and some painkillers, and yet these are substances that doctors are *able to* provide remote prescriptions for. The point is not that healthcare professionals will *always* prescribe such medications remotely (GPs often do where clinical need dictates it), but that they *can* exercise their discretion to determine where and how prescription is appropriate without excessive regulation.

The empirical evidence suggests that the indications about the appropriateness of the drug regimen of EMA that can be gathered from verbal consultation are sufficient to ensure safe administration. Thus, it is difficult to make the case that *physical* examination or face-to-face consultation *before* taking the medications is *generally* necessary. There will, of course, be instances in which it is appropriate for doctors to suggest that a pregnant person needs to physically attend a clinic before receiving a prescription. For example, where the patient’s medical history or the content of a remote consultation indicates a heightened risk of an ectopic pregnancy. However, because the medication is safe and routine treatment, it is not the case that every instance of termination requires this level of supervision. There is no evidence to suggest that prescription of abortion medication need be a matter of law (and *legally mandated*) such that *in every case* a pregnant person *must* receive their prescription and initial treatment in an abortion clinic. To insist on this results in real barriers to access—people often have to travel considerable distances to access clinics,^141^ and this is an obstacle to care for people in remote areas, disabled people, and people with limited income.^142^

These claims regarding the necessity of physical examination/face-to-face consultation are premised on concerns about the safety of prescription, but are all ultimately unfounded. Erdman explains that abortion is ‘often targeted for excessive regulation due to falsehoods about its inherent risks or dangerousness, [which is] a function of abortion stigma. The over-regulation of abortion throughout pregnancy on grounds of medical need or safety is another instance of boundary crossing, where moral and material hazards emerge’.^143^ Though Erdman is writing about the provision of treatment later in pregnancy, her arguments are also important in this context. EMA is safe and insisting that it is singled out as an instance in which remote consultation is inappropriate serves only to stigmatise the process and make access to treatment more difficult.

### B. Can Adequate Information About Mifepristone and Misoprostol Be Provided By Remote Consultation, Or Is Face-To-Face Consultation Necessary?

Healthcare professionals must ensure that adequate information is given regarding medical treatments to enable patients to give their informed consent.^144^ The specifics of adequate informed consent are provided by the Supreme Court ruling in *Montgomery.*^145^ Broadly speaking, there is a requirement on individual doctors in exercising their duty of care to give appropriate information about the treatment at hand, associated risks, and alternative options, to enable the patient to understand what is involved in their treatment and make an informed decision about whether to proceed. This shifted the approach from providing information deemed appropriate by the *reasonable doctor* to addressing the needs of the *reasonable patient* to facilitate informed consent. Professional guidance for doctors on obtaining an appropriate consent is provided by the GMC.^146^ In addition to reinforcing the principles established in *Montgomery*, the GMC urges doctors to try and ascertain what matters to a particular patient in order to individualise the information provided.

It remains a professional matter for the doctor to determine what constitutes a relevant alternative for a reasonable patient.^147^ In this context, the alternatives are limited to two other options. Patients may consider not having the treatment and continuing the pregnancy, assuming their health is not at serious risk from so doing, or they can opt to have a surgical abortion rather than EMA. Neither of these options are sufficiently complex or difficult to understand to mean that the information cannot be conveyed remotely. The key differences between EMA and surgical abortion can be easily explained such that a patient who would prefer surgical abortion—for reasons such as less pain, fewer side effects, and a more definitive procedure—can make this choice, while those who favour the flexibility, privacy, and less invasive nature of EMA can decide in line with their preferences.^148^ This information is largely covered by existing decision aids provided by NICE.^149^ The option of continuing the pregnancy will usually have been carefully considered by the patient before the consultation because rather than being a medical choice between treatment options, it is an obvious choice between two pathways (reproducing or not) that all would be aware of.

With respect to information about the treatment and risks of EMA, NICE guidelines on Abortion Care outline the key information that people must be provided with in order to give their informed consent to medical treatment terminating pregnancy.^150^ This includes specific aspects about self-administration of misoprostol following the 2018 change in law. The guidelines explain that patients must have information to help them prepare for abortion, including what abortion encompasses both during and after and ‘how much pain and bleeding to expect’,^151^ ‘that they may see the products of pregnancy as they are passed’, and ‘what the products of pregnancy will look like and whether there may be movement’.^152^ Patients must be provided with information ‘on signs and symptoms that indicate they need medical help after an abortion and who to contact if they do’,^153^ and ‘information about different options for management and disposal of pregnancy remains’.^154^ There is nothing intrinsic to this information that means it cannot be communicated in and understood during a remote consultation, since it involves a thorough discussion about risks that would involve the same substance over the phone or video-call as it would in person.

HCPs (health care professionals) must work on the assumption that patients have capacity^155^ and so it is highly unlikely that a capacity assessment would be appropriate for a patient who is independently seeking an abortion. It is, however, essential to bear in mind that HCPs are required to check that patients have understood the information. They should be alert to signs that the patient needs additional support to understand and retain information in order to make and communicate a decision.^156^ It is possible that remote consultation makes it more difficult to be sufficiently sure that the patient has understood information. For example, if the consultation is by telephone and the HCP cannot observe visual non-verbal signs that the information is understood, or if there is a poor audio connection and it becomes challenging to communicate. This, however, is a risk that is capable of being addressed in two ways. Video-calling as an alternative to voice calling can be used to enable visual assessment where it is felt necessary, for example, if there is a concern that a patient might be vulnerable or they might not have understood. Evidence from the Australian telemedicine abortion experience suggests that telephone consultation works well,^157^ and video-call should not become mandatory because some clients may not have the appropriate equipment. However, given that access to video-calling technology is rapidly becoming more widely available, it is arguably a useful *optional* tool for remote consultation. Further, potential savings from the increased use of telemedicine may result in savings that can be redistributed to enable access for those without the appropriate technology to video-call.^158^ Whether the initial consultation is done via phone or video, if a HCP is not satisfied that the patient has understood during a remote consultation, or if there is concern about vulnerability or coercion, they can require a face-to-face consultation in clinic in order to ascertain both that informed consent has been achieved and that the patient is not subject to coercion. Thus, remote consultation for EMA should be viewed as a pathway that would be appropriate for *most* patients but not *all*.

There is also evidence to suggest that a conversation by remote consultation might result in a more positive, less stressful experience for people, making it easier for them to engage with the information provided. A remote consultation may be preferable for people who fear that there is stigma attached to abortion, which leads to concern that HCPs might judge them or look down on them for failing to avoid pregnancy. Speaking to a HCP via telephone or video-call may thus be a less stressful experience which promotes privacy and dignity. This is supported by research into remote provision in Australia, which indicates that people associate remote access with greater privacy, as well as more convenient and faster access to treatment.^159^

Remote consultation also removes the challenges of attending a clinic surrounded by protesters, which unfortunately has been the fate of so many seeking abortion. People who have attended a clinic for treatment often report experiencing severe distress because of the presence of protestors. Some have described going into their appointments ‘physically shaking’, feeling harassed, and being in a ‘total state, heart pounding’.^160^ Some people may experience a heightened state of anxiety during the clinic consultation as a result of the stress of arriving and fear about leaving the building after their appointment. This may make it more difficult to take in all the information provided and to engage with the information about the treatment, what will happen and what they should do. If people can receive their consultation at home, where they may feel more comfortable and relaxed, they may find it *easier* to understand and follow information, and to ask questions about the treatment simply because the atmosphere is less tense.

This debate—about remote consultation and prescription—is simply one of the latest in a long parade of wrangles over the law permitting abortion. In one sense, however, this issue is distinct because it speaks to the practicality and experience of accessing services in the twenty-first century, rather than questioning the fundamental approach to allowing people to seek abortion. Healthcare professionals remain involved, and the assessments remain the same. Since there is no empirical reason to overregulate EMA, might there be a less tangible but equally important reason to restrict access? Feintuck explains that public interest values and socio-political forces engage upon broader questions about what is permissible and acceptable.^161^ Thus, considering the appropriate regulatory framework also means engaging with the question of *what* it is that people need to be protected from.^162^ The obvious answer here is politically-motivated concern of EMA being too easy to access, and satisfying the pro-life/anti-choice lobby. Thus, rather than addressing a genuine risk about safety, the restrictive approach allows an unjustifiable obstacle for the gratification of a particular group who—we might assume—would not wish to access abortion services personally but rather seek to curtail the autonomy of others. It seems clear that underlying the excessive medical control of EMA that drastically interferes with clinical discretion is not a matter of necessity at all. As Sheldon has observed, many judgments have been clear that such regulation is not a necessity but a response to a controversial issue.^163^ In a 2011 case challenging the then prohibitions on home use of *both* abortion medications,^164^ Justice Supperstone made reference to the importance of maintaining medical control of abortion—including conditions placed on who can authorise abortions, when, and how—because it is controversial.^165^ Sheldon considers that the reason the abortion pill was singled out as a treatment that could not (at the time of her writing) be self-administered absent supervision was ‘by virtue of the morally sensitive nature of the procedure which distinguishes it from other medical treatments’.^166^

The time has come for change. As Sheldon and others have observed, the view that, ‘abortion should be available to any woman who wants one—has become a wide spread orthodoxy within modern British abortion services, at least in the context of early pregnancy’.^167^ Continuing to demand unnecessary and unjustifiable requirements on those seeking to access EMA is anachronistic and problematic. Those who have not accepted Sheldon and colleagues’ view on abortion ‘orthodoxy’, might maintain that the reason for treating EMA differently relates to the fear that remote access trivialises abortion by making it ‘too easy’. However, it is not clear how travelling to the clinic and having the consultation face-to-face, rather than remotely, changes the fundamental nature of the encounter or the decision that is made. The information exchanged and discussed is the same and the quality of the decision—to continue a pregnancy or not—remains unchanged.

If, on the other hand, the key reason for insisting upon clinic attendance is safety and welfare, these are concerns that require more careful attention and an individualised approach. EMA is a safe treatment provided both in person and remotely. This has been well established by a wealth of evidence collected both during the pandemic in the UK, and before the pandemic in several other countries, such as Australia and the USA. Many who object to remote consultation do so on the grounds that face-to-face consultation is the only way to ensure sufficient safeguarding for individuals.^168^ However, that ‘safeguarding is being centred in the discourse around telemedical abortion reiterates the problematic characterisation of women seeking to end unwanted pregnancies as being inherently vulnerable and in need of institutional support’.^169^ The reality is that safeguarding issues are not relevant in the vast majority of cases, and the objection based on safeguarding is used to reduce access for the majority of people.

We do acknowledge that for a very small minority who might be vulnerable—because of age, health issues, a language barrier, or because they are being coerced—a safeguarding process may be necessary. Adequate safeguarding can be undertaken remotely. Many people in abusive relationships report not being able to attend clinics at all (and thus accessing abortion medications unlawfully online).^170^ And often when abuse victims do attend clinics, they have difficulty disclosing welfare issues for fear of repercussions from an abuser—even when that abuser is not present.^171^ Prohibiting remote consultation on the grounds that safeguarding is inadequate is, ironically,

forcing these [vulnerable] women into a situation entirely devoid of safeguarding. Even if in-person care was considered to be the ideal, remote provision results in fewer [vulnerable] women having no access to care or accessing care unlawfully.^172^

The reality is that the matter is not a case of face-to-face consultation versus remote consultation, but—in lots of cases—remote consultation versus no contact whatsoever with healthcare professionals. Some question whether remote consultation enables abusive partners/family members to interfere; however, even when consultation takes place in-person, an abuser may be present, or prevent the pregnant person attending the clinic at all. While coerced abortion is a concern,^173^ we know from the jurisprudence that reported instances of coerced abortion involve abortifacients obtained unlawfully,^174^ rather than legitimately. Rather than assuming that remote consultation will make coerced abortions more likely to happen, they may serve to prevent coercion.

Remote safeguarding processes are being shown to be effective, with care providers having reported that these processes are being utilised more frequently in the context of TEMA.^175^ Disclosing potential welfare issues may be easier for people when they speak with a healthcare professional over the phone or by video-call—rather than in person. Many people find clinical environments intimidating and find discussing private matters in-person difficult. They may find it easier to disclose potential safeguarding matters remotely when they can be in a space they find comfortable.^176^ Healthcare professionals should be trained to spot signs of concern during a consultation (e.g., change in strength or tone of voice).^177^ In some instances healthcare professionals may decide—on the basis of concerns about the quality of consultation, or the potential presence of a coercive individual—that they want to exercise their discretion to ask the patient to attend clinic.^178^ Our point is that face-to-face consultation for EMA should be a matter of *clinical discretion (*and/or patient preference)^179^—as is the case in the prescription of all other prescription-only medicines—as opposed to being a *mandatory* requirement for lawful abortion.

### C. Does It Need to Be a Doctor Who Prescribes?

Empirical evidence demonstrates that health outcomes are just as good when nurses prescribe.^180^ Moreover, nurse prescription may improve health outcomes because it might reduce waiting times.^181^ It is clear that nurses are qualified to prescribe EMA (even though they are not legally permitted to do so) within the current system. Nurses with the qualifications to prescribe medications in general could also prescribe mifepristone in other contexts (where use of the medications is not *for* abortion) as the regulation is not specifically permitting anyone but a doctor prescribing this medication—which does have other uses; it is only where prescription is intended to be *for* abortion that it comes under the purview of the legislation. Furthermore, the way service providers are organised in the UK and to enable task sharing for efficient service provision (recommended by the WHO^182^, NICE,^183^ and the Royal College of Obstetricians and Gynaecologists)^184^ often means that nurse-midwives are the people who undertake consultations with patients, and doctors issue prescriptions by reviewing the information that they have collected.^185^ Department of Health guidance recognises this and specifies that this meets the requirements of the AA 1967 provided the doctor that issues the prescription reviews the information collected so they can justify how they ‘reached their decision [to prescribe] in good faith’.^186^ Since nurses are responsible for collecting the information upon which doctors base their decision, surely if it was unsafe for them to prescribe, conducting the consultation would also be unsafe? If they are not qualified to prescribe, then they are arguably not qualified to conduct a consultation and determine what information it is appropriate to collect from the patient. This is recognised by the GMC who recommend that ‘if you delegate the assessment of a patient’s suitability for a medicine, you must be satisfied that the person you delegate to has the qualifications, experience, knowledge and skills to make the assessment’.^187^

Nurses are generally considered qualified to prescribe prescription-only medications.^188^ Distinguishing EMA as drugs nurses cannot prescribe is arbitrary, excessive, and does not protect people’s health. All the current prohibition on nurse prescribing does is add unnecessary bureaucracy. This creates delays in access to care—that are felt by patients—and prevents service providers from adapting in line with medical best practice,^189^ without improving the safety of EMA administration. This is another example of excessive interference with service provision that is not grounded in any immediate necessity to mitigate risk. Nurse prescription has recently been permitted in Northern Ireland,^190^ by regulations produced by Westminster, which we hope is indicative of future change in Great Britain.

## V. CONCLUSION

Abortion is essential healthcare. UK law, however, does not appropriately reflect this. The law surrounding pharmaceuticals designates medications as ‘prescription-only’ to ensure that they are available to patients only when safe and clinically appropriate. Doctors are installed as gatekeepers of medications, but only in so far as it relates to *clinical* factors, and their discretion in prescribing is not further interfered with by the law. In contrast, the law enables excessive state interference into clinical discretion in *abortion*, including imposing conditions about when, where, and by whom EMA can be provided. These regulations are not grounded in clinical necessity, but instead reflect the reality that abortion is exceptionalised. The framing of the law perpetuates abortion stigma.^191^ The legal framework causes harm in creating barriers to access. Excess regulation results in delays to care and prevents service providers from adapting provision in line with best practice to improve efficiency^192^ and improve health. Substantive reform is necessary to reflect the reality that abortion is essential healthcare.

In this article, we noted ways in which changes to the regulation of EMA are necessary, including, making remote prescription and home use of abortion medications permanent in Great Britain and bringing this to Northern Ireland, and enabling lawful prescription by healthcare professionals besides doctors. However, rather than patchwork amendment to the law—for example, the issuing of new permanent approval orders in Great Britain for home use, or amendment to the AA 1967 to allow prescribing by medical *professionals* rather than *practitioners—*broader change is necessary. The case for the decriminalisation of abortion has been made persuasively elsewhere.^193^ As Herring and others observe, ‘it is important to make robust provision within abortion services for informed consent, confidentiality, safeguarding and access to counselling for those women who want it, the current criminal law framework plays no role in this regard’.^194^ Were (early) abortion decriminalised, and decriminalised without the introduction of comprehensive regulation as in Northern Ireland, the law would not routinely interfere with the evolution of medical best practice.^195^ Without additional criminal prohibition on abortion medications, they would remain available only following prescription by a healthcare professional. Healthcare professionals are better placed than politicians to determine the appropriate conditions for prescribing, and to exercise discretion to ensure patient safety. There is no justification, medical or ethical, for mifepristone and misoprostol being subject to more stringent regulation than other prescription-only medications. This bolsters the case for decriminalisation of abortion.

## Footnotes

1 We define *early* medical abortion as medication abortion administered within the first 10 weeks of pregnancy.

2 Department of Health and Social Care, ‘Abortion Statistics, England and Wales: 2019’ (11 June 2020) <https://assets.publishing.service.gov.uk/government/uploads/system/uploads/attachment_data/file/891405/abortion-statistics-commentary-2019.pdf> accessed 28 April 2021, 13. This is the figure for all medical abortions the statistics do not specify how many are *early* medical abortions. In all, 82% of total abortions were performed at less than 10 weeks’ gestation (eleven to twelve), so it is reasonable to assume that the majority of the 73% of medical abortions were *early* medical abortions.

3 Method and gestational age are reported separately. The majority of terminations (8 in 10) are performed in the first 10 weeks. Public Health Scotland, ‘A National Statistics release for Scotland Termination of pregnancy Year ending December 2019’ (25 May 2021) <https://beta.isdscotland.org/find-publications-and-data/population-health/sexual-health/termination-of-pregnancy-statistics/> accessed 28 April 2021.

4 The World Health Organization, ‘Safe Abortion: Technical and Policy Guidance for Health Systems’ (2012) <https://apps.who.int/iris/bitstream/handle/10665/70914/9789241548434_eng.pdf?sequence=1> accessed 7 April 2021. The WHO recommends the mifepristone-misoprostol regimen between nine and 12 weeks’, though on a weaker recommendation based on a lower quality of evidence. There have, however, been many studies since that demonstrate the safety, effectiveness, and acceptability of EMA in this period.

5 EG Raymond and others, ‘First-trimester Medical Abortion with Mifepristone 200 mg and Misoprostol: A Systematic Review’ (2013) 87 Contraception 26; MJ Chen and MD Creinin, ‘Mifepristone with Buccal Misoprostol for Medical Abortion: A Systematic Review’ (2015) 126 Obstetrics & Gynecology 12.

6 RJ Gomperts and others, ‘Using Telemedicine for Termination of Pregnancy with Mifepristone and Misoprostol in Settings Where There is no Access to Safe Services’ (2008) 115 British Journal of Obstetrics and Gynaecology 1171.

7 P Lohr and others, ‘How Would Decriminalisation Affect Women’s Health?’ in S Sheldon and K Wellings (eds), *Decriminalising Abortion in the UK: What Would It Mean?* (Policy Press 2020) 41.

8 Abortion (Northern Ireland) (No 2) Regulations 2020.

9 E Janiak and AB Goldberg, ‘Eliminating the Phrase “Elective Abortion”: Why Language Matters’ (2016) 93 Contraception 89.

10 Lohr and others (n 7) 43.

11 J Keown, *Abortion, Doctors and the Law: Some Aspects of the Legal Regulation of Abortion in England from 1803 to 1982* (Cambridge University Press 1988).

12 JA Parsons and EC Romanis, *Early Medical Abortion, Equality of Access, and the Telemedical Imperative* (OUP 2021) 23.

13 The majority of pregnant people identify as women; however, we use gender-neutral to be inclusive of those people who need access to abortion services, but who do not identify as women.

14 E Jackson, ‘Abortion, Autonomy and Prenatal Diagnosis’ (2000) 9 Social & Legal Studies 463; S Sheldon, ‘The Decriminalisation of Abortion: An Argument for Modernisation’ (2015) 36 Oxford Journal of Legal Studies 334; S Sheldon and K Wellings (eds), *Decriminalising Abortion in the UK: What Would It Mean?* (Policy Press 2020).

15 Notable exceptions: S Sheldon, ‘The Medical Framework and Early Abortion in the UK: How Can a State Control Swallowing?’ in R Cook and others (eds), *Abortion Law in Transnational Perspective* (University of Pennsylvania Press 2014); Lohr and others (n 7); Parsons and Romanis (n 12).

16 E Raymond and others, ‘Sixteen Years of Overregulation: Time to Unburden Mifeprex’ (2017) 376 New England Journal of Medicine 790; A Ahmed, ‘How the COVID-19 Response is Altering the Legal and Regulatory Landscape on Abortion’ (2020) 7 Journal of Law and the Biosciences doi.org/10.1093/jlb/lsaa012.

17 While abortion is criminal most women do not experience the stigma of its criminal status because of provider’s efforts. There is therefore a ‘socio-legal gap’: E Lee, ‘Tensions in the Regulation of Abortion in Britain’ (2003) 30 Journal of Law and Society 532, 553. There may be less of a gap when looking at EMA—people do experience the ramifications of overregulation e.g., in not being able to decide where to take abortion medications.

18 E Jackson, *Regulating Reproduction: Law, Technology and Autonomy* (Hart Publishing 2001) 72.

19 ibid 77.

20 DA Grimes and others, ‘Unsafe Abortion: The Preventable Pandemic’ (2006) 368 Lancet 1908, 1917.

21 Lohr and others (n 7) 38.

22 ibid 39.

23 United Nations, ‘Report of the International Conference on Population and Development’ (1995), <https://www.un.org/development/desa/pd/sites/www.un.org.development.desa.pd/files/icpd_en.pdf> accessed 23 August 2021, para 7.2.

24 World Health Organization (n 4) 1.

25 M Manian, ‘The Consequences of Abortion Restrictions for Women’s Healthcare’ (2014) 71 Washington and Lee Law Review 1317.

26 Janiak and Goldberg (n 9) 91.

27 Royal College of Obstetricians and Gynaecologists and The Faculty of Sexual & Reproductive Healthcare, ‘RCOG and FSRH Key Messages on Safe Abortion’ (1 March 2021) <https://www.rcog.org.uk/globalassets/documents/global-network/projects-and-partnerships/making-abortion-safe/rcog-and-fsrh-key-messages-on-safe-abortion.pdf> accessed 23 August 2021, 3.

28 Alternatives to misoprostol, such as gemeprost, are sometimes used, though rarely.

29 World Health Organization (n 4) 45–46.

30 A 2013 systematic review found that oral administration of misoprostol can result in a higher risk of abortion failure than non-oral routes: Raymond and others (n 5) 30–31.

31 L Wedisinghe and D Elsandabesee, ‘Flexible Mifepristone and Misoprostol Administration Interval for First-Trimester Medical Termination’ (2010) 81 Contraception 269; K Shaw and others, ‘Mifepristone-Misoprostol Dosing Interval and Effect on Induction Abortion Times’ (2013) 121 Obstetrics & Gynecology 1335.

32 M Creinin and others, ‘Mifepristone and Misoprostol Administered Simultaneously Versus 24 Hours Apart for Abortion’ (2007) 109 Obstetrics & Gynecology 885.

33 World Health Organization (n 4).

34 World Health Organization, ‘WHO Model List of Essential Medicines’ (2019) <https://www.who.int/publications/i/item/WHOMVPEMPIAU2019.06> accessed 4 May 2021, 47. The List includes a caveat when referring to the use of misoprostol in combination with mifepristone (meaning where it is used to terminate a pregnancy)—‘[w]here permitted under national law and where culturally acceptable’.

35 Raymond and others (n 17) 28–29.

36 M Chen and M Creinin, ‘Mifepristone with Buccal Misoprostol for Medical Abortion’ (2015) 126 Obstetrics & Gynecology 12; H Bracken and others, ‘Alternatives to Routine Ultrasound for Eligibility Assessment Prior to Early Termination of Pregnancy with Mifepristone-Misoprostol’ (2011) 118 British Journal of Obstetrics and Gynaecology 17.

37 Chen and Creinin (n 36) 18–19.

38 T Ngo and others, ‘Comparative Effectiveness, Safety and Acceptability of Medical Abortion at Home and in a Clinic: A Systematic Review’ (2011) 89 Bulletin of the World Health Organization 360, 365.

39 E Tousaw and others, ‘“It Is Just Like Having a Period with Back Pain”: Exploring Women’s Experiences with Community-Based Distribution of Misoprostol for Early Abortion on the Thailand-Burma Border’ (2018) 97 Contraception 122, 124.

40 K LaRoche and A Foster, ‘“It Gives You Autonomy Over Your Own Choices”: A Qualitative Study of Canadian Abortion Patients’ Experiences with Mifepristone and Misoprostol’ (2020) 102 Contraception 61, 63.

41 Defined in Human Medicines Regulations 2012, reg 2.

42 These medications are medicinal products because they are substances used or administered to restore, correct, or modify a physiological function by exerting a pharmacological action for the purposes of the Human Medicines Regulations 2012, reg 2(b).

43 J Flanigan, ‘Three Arguments against Prescription Requirements’ (2012) 28 Journal of Medical Ethics 579, 580.

44 E Jackson, *Law and the Regulation of Medicines* (Bloomsbury Publishing 2013) 3.

45 ibid 3.

46 Human Medicines Regulations 2012, reg 217(3)(a).

47 ibid reg 217(2).

48 Defined as a signature that is uniquely linked to the practitioner that identifies them, is created by a means that only the practitioner can control and any subsequent change to it would be detectable—see ibid reg 218(5).

49 ibid reg 219(2).

50 There are additional requirements for medications regulated by the Misuse of Drugs Act 1977 and the Misuse of Drugs Regulations 2011 as ‘controlled substances’—mifepristone is not one of these medications. Notably, none of these requirements interfere with a practitioner’s decision making about *when* to prescribe controlled substances.

51 Offences Against the Person Act 1861, s 59.

52 K Norrie, ‘Abortion in Great Britain: One Act, Two Laws’ (1985) Criminal Law Review 475.

53 Abortion Act 1967, s 1.

54 ibid.

55 ibid s 3.

56 ibid s7(3).

57 M Fox and G Horgan, ‘The Effects of Decriminalisation in Northern Ireland’ in S Sheldon and K Wellings (eds), *Decriminalising Abortion in the UK: What Would It Mean?* (Policy Press 2020).

58 s 9.

59 Abortion (Northern Ireland) (No 2) Regulations 2020, reg 11.

60 ibid, though reg 11(2) specifies that this offence is not committed by ‘the woman herself’.

61 As amended by Human Fertilisation and Embryology Act 1990, s 37.

62 ibid.

63 Abortion Act 1967, s 1(2).

64 Jackson (n 18) 80.

65 M Brazier and E Cave, *Medicine, Patients and the Law* (Manchester University Press 2016) 404.

66 ibid.

67 Parsons and Romanis (n 12) 16.

68 EG Raymond and DA Grimes, ‘The Comparative Safety of Legal Induced Abortion and Childbirth in the United States’ (2012) 119 Obstetrics & Gynecology 215. This study compared childbirth with legal induced abortion (including methods other than EMA). However, given the 14-fold difference in the risk of death reported, alongside the previously discussed data on the safety of EMA, it is reasonable to take these findings as applicable to EMA.

69 Abortion (Northern Ireland) (No 2) Regulations 2020, reg 4(1)(a)-(b); reg 5 provides a ground for abortion where immediately necessary to save the pregnant person’s life; reg 6 where there is a risk to the pregnant person’s life or of grave, permanent injury; and reg 7 where there is a severe foetal abnormality.

70 Parsons and Romanis (n 12) 23.

71 (Emphasis added); *Royal College of Nursing of the United Kingdom v Department of Health and Social Security* [1981] 1 All ER 545, 569–70 (Lord Diplock).

72 National Institute of Health and Care Excellence. ‘Abortion Care [NG140]’ (25 September 2019) <https://www.nice.org.uk/guidance/ng140/> accessed April 2021, para 1.1.11.

73 House of Commons Science and Technology Committee, ‘Scientific Developments Relating to the Abortion Act 1967’ <https://publications.parliament.uk/pa/cm200607/cmselect/cmsctech/1045/1045i.pdf> accessed 30 April 2021, para 89.

74 EC Romanis and JA Parsons, ‘Legal and Policy Responses to the Delivery of Abortion Care during COVID‐19’ (2020) 151 International Journal of Gynecology and Obstetrics 479, 482.

75 The Abortion (Northern Ireland) (No 2) Regulations 2020, reg 3.

76 ibid reg 2(2)(a)-(c).

77 Explanatory Memorandum to the Abortion (Northern Ireland) (No 2) Regulations 2020, para 7.18.

78 JA Parsons and EC Romanis, ‘2020 Developments in the Provision of Early Medical Abortion by Telemedicine in the UK’ (2021) 125 Health Policy 17.

79 ibid 21. Consultations were conducted separately in Scotland, England, and Wales. Only Scotland has published initial findings. Scottish Government, ‘Early Medical Abortion at Home - Future Arrangements: Consultation Analysis’ (23 June 2021) <https://www.gov.scot/publications/future-arrangements-early-medical-abortion-home-consultation-analysis/> accessed 23 August 2021.

80 Abortion Act 1967, ss 1(3) and (3A); *R (on the application of Christian Concern) v Secretary of State for Health and Social Care* [2020] EWCA Civ 1239 at [55].

81 Abortion Act 1967, ss 1 (3) and (3A), as amended by Human Fertilisation and Embryology Act 1990, s 37.

82
*SPUC Pro-Life Scotland Limited v Scottish Ministers* [2018] CSOH 85.

83
*R (on the application of Christian Concern)* (n 80), at [55].

84 Where doctors are prosecuted under the Offences Against the Person Act, few juries convict and judges encourage hesitancy in their direction, stating that there must be clear evidence of bad faith: See *Paton v British Pregnancy Advisory Service* [1979] QB 276.

85 The Abortion (Northern Ireland) (No 2) Regulations 2020, reg 8(4).

86 ibid reg 11.

87 Department of Health and Social Care, ‘The Abortion Act 1967 – Approval of a Class of Places’ (27 December 2018) <https://assets.publishing.service.gov.uk/government/uploads/system/uploads/attachment_data/file/768059/Approval_of_home_use_for_the_second_stage_of_early_medical_abortion.pdf> accessed 15 April 2021.

88 ibid.

89 Scottish Minister for Public Health, ‘The Abortion Act 1967 (Place for Treatment for the Termination of Pregnancy) (Approval) (Scotland)’ (26 October 2017) <https://www.sehd.scot.nhs.uk/cmo/CMO(2017)14.pdf> accessed 15 April 2021.

90 Welsh Minister for Health and Social Services, ‘The Abortion Act 1967 (Approval of Place for Treatment for the Termination of Pregnancy) (Wales)’ (20 June 2018) <https://gov.wales/sites/default/files/publications/2019-07/the-abortion-act-1967-approval-of-place-for-treatment-for-the-termination-of-pregnancy-wales-2018-2018-no-56.pdf> accessed 15 April 2021.

91
*SPUC Pro-Life Scotland Limited v Scottish Ministers* (n 82).

92 National Institute of Health and Care Excellence (n 72) para 1.1.9.

93 The Abortion (Northern Ireland) (No 2) Regulations 2020, reg 8(6).

94 ibid reg 8(2)(a)-(c).

95 Northern Ireland (Executive Formation, etc) Act 2019, s 9.

96 Fox and Horgan (n 57).

97 The Abortion (Northern Ireland) (No 2) Regulations 2020, reg 11(2).

98 ibid reg 11(1).

99 EC Romanis and others, ‘COVID-19 and Reproductive Justice: and Reproductive Justice in Great Britain and the United States: Ensuring Access to Abortion Care during a Global Pandemic’ (2020) 7 Journal of Law and the Biosciences https://doi.org/10.1093/jlb/lsaa027.

100 Department of Health and Social Care, ‘The Abortion Act 1967 – Approval of a Class of Places’ (30 March 2020) <https://assets.publishing.service.gov.uk/government/uploads/system/uploads/attachment_data/file/876740/30032020_The_Abortion_Act_1967_-_Approval_of_a_Class_of_Places.pdf> accessed 12 April 2021.

101 Welsh Government, ‘The Abortion Act 1967—Approval of a Class of Place for Treatment for the Termination of Pregnancy (Wales) 2020’ (31 March 2020) <https://gov.wales/sites/default/files/publications/2020-04/approval-of-a-class-of-place-for-treatment-for-the-termination-of-pregnancy-wales-2020.pdf> accessed 15 April 2021.

102 Scottish Government, ‘The Abortion Act 1967 (Place for Treatment for the Termination of Pregnancy) (Approval) (Scotland) 2020’ (31 March 2020) <https://www.sehd.scot.nhs.uk/cmo/CMO(2020)09.pdf> accessed 15 April 2021.

103 ibid—it is possible that the order could remain in place far longer than those in England and Wales. It is notable that TEMA will not *automatically* revert to being unlawful once pandemic restrictions are over: Parsons and Romanis (n 78) 19.

104 Parsons and Romanis (n 12) 138.

105 ibid.

106 Scottish Government (n 102).

107 Romanis and others (n 99); Parsons and Romanis (n 12) 40.

108 Lohr and others (n 7) 51.

109 The Abortion (Northern Ireland) (No 2) Regulations 2020, reg 8(3).

110 The BPAS website states that the service is for people in Northern Ireland less than 10 weeks pregnant and who ‘cannot leave home because they are shielding or self-isolating because they or someone in their household has COVID-19 symptoms’: British Pregnancy Advisory Service, ‘Abortion pills by post for women from Northern Ireland’ (2020) <https://www.bpas.org/abortion-care/considering-abortion/northern-ireland-pills-by-post/> accessed 25 April 2021.

111 Flanigan (n 43) 580.

112 J Erdman and R Cook, ‘Decriminalization of Abortion – A Human Rights Imperative’ (2020) 62 Best Practice & Research Clinical Obstetrics & Gynaecology 11, 14.

113 Parsons and Romanis (n 12) 115.

114 A Aiken and others, ‘Effectiveness, Safety and Acceptability of No‐Test Medical Abortion (Termination of Pregnancy) Provided via Telemedicine: a National Cohort Study’ (2021) 128 BJOG 1464–74.

115 ibid 1469.

116 ibid 1469.

117 J Reynolds-Wright and others, ‘Telemedicine Medical Abortion at Home under 12 Weeks’ Gestation: a Prospective Observational Cohort Study during the COVID-19 Pandemic’ (2021) BMJ Sexual and Reproductive Health, doi:10.1136/bmjsrh-2020-200976, 3.

118 E.g., M Endler and others, ‘Telemedicine for Medical Abortion: A Systematic Review’ (2019) 126 BJOG 1094.

119 Parsons and Romanis (n 78).

120 General Medical Council, ‘Remote Consultations’ <https://www.gmc-uk.org/ethical-guidance/ethical-hub/remote-consultations> accessed 6 February 2020.

121 General Medical Council, ‘Good Practice in Prescribing and Managing Medicines and Devices,’ (March 2013) <https://www.gmc-uk.org/-/media/documents/prescribing-guidance_pdf-59055247.pdf> accessed 6 February 2020, 9. This guidance was withdrawn on 5 April 2021.

122 ibid 9.

123 General Medical Council, ‘Good Practice in Prescribing and Managing Medicines and Devices’ (5 April 2021), <https://www.gmc-uk.org/-/media/documents/prescribing-guidance-updated-english-20210405_pdf-85260533.pdf?la=en&hash=716B06E30FA2D9CA7700B94B3F55173B10F3058A> accessed 26 May 2021.

124 ibid 5.

125 GMC guidance only mentions one specific instance in which it is necessary to prescribe a specific medication face-to-face- cosmetic injectables—ibid 5; General Medical Council (n 123) 9.

126 S Bullo, ‘“I Feel Like I’m Being Stabbed by a Thousand Tiny Men”: The Challenges of Communicating Endometriosis Pain’ (2020) 24 Health 476.

127 R Jones and H Boonstra, ‘The Public Health Implications of the FDA Update to the Medication Abortion Label’ (2016) <https://www.guttmacher.org/article/2016/06/public-health-implications-fda-update-medication-abortion-label> accessed 12 April 2021.

128 Scottish Government, ‘Consultation on Future Arrangements for Early Medical Abortion at Home: Response 629028760’ <https://consult.gov.scot/population-health/early-medical-abortion-at-home/consultation/view_respondent?uuId=629028760> accessed 23 August 2021.

129 Bracken and others (n 36) 20.

130 ibid.

131 ibid.

132 D Schonberg and others, ‘The Accuracy of Using Last Menstrual Period to Determine Gestational Age for First Trimester Medication Abortion: A Systematic Review’ (2014) 90 Contraception 480.

133 Aiken and others (n 114).

134 Parsons and Romanis (n 12) 93.

135 Society for the Protection of Unborn Children, ‘The Case Against DIY Abortion’ (2020) <https://www.spuc.org.uk/Get-Involved/Campaigns/The-Case-Against-DIY-Abortions> accessed 12 May 2021.

136 Jackson (n 44) 94.

137 ibid 94.

138 A Aiken and others, ‘Barriers to Accessing Abortion Services and Perspectives on using Mifepristone and Misoprostol at Home in Great Britain’ (2018) 97 Contraception 177.

139 British Pregnancy Advisory Service, ‘Abortion: Frequently asked Questions: How Confidential is the Service?’ (2021) <https://www.bpas.org/abortion-care/considering-abortion/abortion-faqs/> accessed 12 May 2021; British Pregnancy Advisory Service, ‘Health Records: What we do with your Information’ (2021) <https://www.bpas.org/about-our-charity/governance/health-records/> accessed 12 May 2021.

140 E Milne, ‘Banning Safe Home-Use Abortion Pills Will Leave More Women in Crisis’ (2021) <https://theconversation.com/banning-safe-home-use-abortion-pills-will-leave-more-women-in-crisis-154594> accessed 1 May 2021.

141 Aiken and others (n 114).

142 Romanis and others (n 99).

143 J Erdman, ‘Theorizing Time in Abortion and Human Rights’ (2017) 19 Health and Human Rights Journal 29, 34.

144
*Montgomery v Lanarkshire Health Board* (2015) UKSC 11.

145 ibid.

146 General Medical Council, ‘Decision Making and Consent’ (2020) <https://www.gmc-uk.org/-/media/documents/gmc-guidance-for-doctors–-decision-making-and-consent-english_pdf-84191055.pdf?la=en&hash=BE327A1C584627D12BC51F66E790443F0E0651DA> accessed 1 May 2021.

147 E Cave and C Milo, ‘Informing Patients: The *Bolam* Legacy’ (2020) 20 Medical Law International 103, 112.

148 Parsons and Romanis (n 12) 11.

149 National Institute of Health and Care Excellence, ‘Abortion before 14 weeks: Choosing Between Medical or Surgical Abortion Decision Aid’ (2019) <https://www.nice.org.uk/guidance/ng140/resources/abortion-before-14-weeks-choosing-between-medical-or-surgical-abortion-patient-decision-aid-pdf-6906582255> accessed 4 May 2021.

150 National Institute of Health and Care Excellence (n 72).

151 ibid para 1.2.7.

152 ibid.

153 ibid para 1.2.9.

154 ibid para 1.2.10.

155 Mental Capacity Act 2005.

156 E.g., General Medical Council (n 121); (n 123).

157 P Hyland and others, ‘A Direct-to-Patient Telemedicine Abortion Service in Australia; Retrospective Analysis of the First Eighteen Months’ (2018) 58 Australian and New Zealand Journal of Obstetrics and Gynaecology 335.

158 JA Parsons, ‘The Telemedical Imperative’ (2021) 35 Bioethics 298, 300.

159 L Fix and others, ‘At Home Telemedicine for Medical Abortion in Australia: A Qualitative Study of Patient Experiences and Recommendations’ (2020) 46 BMJ Sexual and Reproductive Health 172.

160 Back Off Campaign, <https://back-off.org/> accessed 1 May 2021.

161 M Feintuck, ‘Precautionary Maybe, but What’s the Principle? The Precautionary Principle, the Regulation of Risk and the Public Domain’ (2005) 32 Journal of Law and Society 271, 372.

162 ibid 393.

163 Sheldon (n 15) 208.

164
*British Pregnancy Advisory Service v Secretary of State for Health* [2011] EWHC 235 (Admin).

165 ibid at [2]; Sheldon (n 15) 208.

166 ibid 203.

167 S Sheldon and others, ‘The Abortion Act (1967): A Biography’ (2019) 39 Legal Studies 18, 33.

168 Society for the Protection of Unborn Children (n 135); See also the Parliamentary debates surrounding remote abortion care: HC Deb 6 July 2020, vol 678.

169 EC Romanis and others, ‘Safeguarding and Teleconsultation for Abortion,’ (2020) 398 The Lancet 555, 556.

170 Aiken and others (n 114) 181.

171 Romanis and others (n 169) 556.

172 ibid 557.

173 E.g., *R (on the application of Christian Concern)* (n 80) at [23].

174 E.g., *R v Magira* [2008] EWCA Crim 1939.

175 M Nevill and K Hills, ‘Safeguarding and Telemedical Abortion Services’ (2021) BMJ Sexual and Reproductive Health, *online first* doi:10.1136/bmjsrh-2020-200891.

176 Romanis and others (n 169) 556.

177 ibid 556.

178 ibid; Nevill and Hills (n 175).

179 Parsons and Romanis (n 12) 108–9.

180 H Kopp Kallner and others, ‘The Efficacy, Safety and Acceptability of Medical Termination of Pregnancy Provided by Standard Care by Doctors or by Nurse‐Midwives: A Randomised Controlled Equivalence Trial’ (2014) 122 BJOG 510.

181 Romanis and Parsons (n 74) 481.

182 World Health Organization, ‘WHO Recommendations: Optimizing Health Worker Roles to Improve Access to Key Maternal and Newborn Health Interventions Through Task Shifting’ (2012) <https://apps.who.int/iris/bitstream/handle/10665/77764/9789241504843_eng.pdf;jsessionid=66FC87E14F80706B491D42220E3019AE?sequence=1> accessed 1 May 2021.

183 National Institute of Health and Care Excellence (n 72) para 1.1.11.

184 Royal College of Obstetricians and Gynaecologists and The Faculty of Sexual & Reproductive Healthcare (n 27).

185 Romanis and Parsons (n 74) 482.

186 Department of Health, ‘Guidance in Relation to Requirements of the Abortion Act 1967’ (2014) <https://assets.publishing.service.gov.uk/government/uploads/system/uploads/attachment_data/file/313459/20140509_-_Abortion_Guidance_Document.pdf> accessed 1 May 2021, 9.

187 General Medical Council (n 123) 10.

188 Human Medicines Regulations 2012, reg 2.

189 Lohr and others (n 7) 43.

190 Abortion (Northern Ireland) (No 2) Regulations 2020, reg 2(2).

191 R Cook, ‘Stigmatized Meanings of Criminal Abortion Law’ in R Cook and others (eds), *Abortion Law in Transnational Perspective* (University of Pennsylvania Press 2014); Sheldon (n 14).

192 Lohr and others (n 7).

193 See n 14.

194 J Herring and others, ‘Would Decriminalisation Mean Deregulation?’ in S Sheldon and K Wellings (eds), *Decriminalising Abortion in the UK: What Would It Mean?* (Policy Press 2020) 62.

195 Lohr and others (n 7).

